# Case report: One-stage craniectomy and cranioplasty digital workflow for three-dimensional printed polyetheretherketone implant for an extensive skull multilobular osteochondosarcoma in a dog

**DOI:** 10.3389/fvets.2024.1459272

**Published:** 2024-08-29

**Authors:** Marc Hobert, Neha Sharma, Caroline Benzimra, Sandro Hinden, Anna Oevermann, Michaela Maintz, Michel Beyer, Florian Thieringer, Julien Guevar

**Affiliations:** ^1^AniCura Tierklinik Thun, Thun, Switzerland; ^2^Oral and Cranio-Maxillofacial Surgery, University Hospital Basel, Basel, Switzerland; ^3^Medical Additive Manufacturing Research Group (Swiss MAM), Department of Biomedical Engineering, University of Basel, Allschwil, Switzerland; ^4^Vetpixel SAS, Kochersberg, France; ^5^Division of Neurological Sciences, DCR-VPH, Vetsuisse Faculty, University of Bern, Bern, Switzerland; ^6^Institute for Medical Engineering and Medical Informatics IM^2^, University of Applied Sciences and Arts Northwestern Switzerland, Muttenz, Switzerland

**Keywords:** craniotomy, craniectomy, PEEK-polyether ether ketone, multilobular osteochondroma, dog

## Abstract

**Objective:**

To report a digital workflow for use and long-term outcome of cranioplasty with a 3D-printed patient-specific Polyetheretherketone (PEEK) implant in a 12-y-old German Shepherd dog after surgical removal of an extensive occipital bone multilobular osteochondrosarcoma (MLO).

**Study design:**

Retrospective case report.

**Animal:**

A 12-year-old neutered female German Shepherd dog was presented with facial deformity, blindness, tetraparesis, and ataxia. Magnetic resonance imaging (MRI) and computed tomography (CT) identified a large skull-based mass extending extra-and intracranially with severe compression of the cerebellum and occipital lobes of the cerebrum.

**Methods:**

One-stage decompressive craniectomy using virtual surgical planned 3D-printed craniotomy cutting guides and the Misonix BoneScalpel^®^ and reconstruction with a patient-specific 3D-printed PEEK cranial implant.

**Results:**

3D-printed craniectomy cutting guides allowed an adequate fit of the cranial implant to the original skull. Misonix BoneScalpel^®^ allowed performing a safe and extensive craniectomy. Postoperative CT (8 weeks after surgery) confirmed the PEEK cranial implant to be in place and without implant rejection. Clinically, the neurological examination identified only a right-hind limb delay in proprioception 8 weeks postoperatively, which remained unchanged at 18 months after surgery. Adjunctive treatment included metronomic chemotherapy. Eighteen months after surgery the dog passed away for reasons unrelated to the MLO, no implant-related complications were reported.

**Conclusion:**

3D-printed craniectomy cutting guides, patient-specific PEEK cranial implant, and metronomic chemotherapy can lead to a successful long-term outcome in dogs with extensive skull MLO.

**Clinical significance:**

PEEK is an alternative biomaterial that can be used successfully for skull reconstruction.

## Introduction

Multilobular osteochondrosarcoma (MLO) is a prevalent and aggressive primary bone tumor affecting the flat bones of the canine skull. Characterized by rapid growth and local invasion, MLO often presents with facial asymmetry and neurological deficits due to significant mass effects on the underlying brain parenchyma. Diagnosis relies on advanced imaging techniques like computed tomography (CT) and magnetic resonance imaging (MRI) (1), often supplemented by fine-needle aspiration or biopsy.

Surgical intervention remains the mainstay of MLO treatment (2), aiming for complete tumor resection to maximize long-term survival and minimize recurrence risk. Additionally, decompressive craniectomy is frequently employed to alleviate intracranial pressure (ICP) and mitigate neurological complications associated with MLO’s rapid expansion. Following extensive tumor removal, substantial cranial defects necessitate cranioplasty for cranial reconstruction and brain protection. Various reconstruction techniques have been used for dogs (3–10).

This case report describes the successful management of MLO in a canine patient implementing a digital workflow utilizing two innovative surgical techniques: virtual surgical planning (VSP) and medical 3D printing. VSP allows for meticulous preoperative planning, facilitating precise tumor resection and minimizing the risk of iatrogenic complications. Furthermore, medical 3D printing enables the creation of patient-specific cutting guides and implants, tailored to the individual anatomy of the patient (11–13).

The use of 3D-printed cutting guides offers enhanced surgical precision, ensuring accurate execution of the preoperative plan and reducing intraoperative variability (14). Additionally, a 3D-printed patient-specific polyetheretherketone (PEEK) cranial implant was utilized in this case. PEEK, a biocompatible material, presents several advantages for cranioplasty, including excellent biocompatibility, radiolucency (adequate for MRI), compatibility with radiation therapy and mechanical properties resembling cortical bone (15, 16).

To the author’s knowledge, this is the first case report in the scientific literature regarding the digital workflow for PEEK 3D printing for cranioplasty in veterinary medicine. By incorporating these advancements, this case report contributes to the growing body of evidence supporting the utilization of advanced surgical techniques in improving MLO management and enhancing patient outcomes in veterinary oncology. This approach can improve surgical precision, optimize implant fit, and potentially reduce complication rates compared to conventional methods.

## Clinical report

### History, clinical evaluation, and findings

A 12 year old, neutered Female German Shepherd dog was presented with a chronic, progressively worsening history of decreased alertness, stumbling on all four limbs, and blindness. Physical examination revealed a facial deformity with a bilobed mass in the occipital region. The neurological evaluation identified diminished alertness and awareness, with absent menace responses bilaterally. Gait analysis demonstrated hypermetria with tetraparesis and ataxia affecting all four limbs. All spinal reflexes, including the cutaneous trunci muscle reflex, were normal. Vertebral column manipulation and palpation were unremarkable. No head tilt, nystagmus, or wide-based stance were observed. Complete blood count and serum biochemistry results fell within normal reference ranges. A multifocal neurological disorder affecting the cerebellum and forebrain was suspected.

MRI and CT imaging datasets of the brain were performed using a 0.25 Tesla magnet (VET MR Grande, Esaote, Genova, Italy) and a 16-slice CT scanner (Somatom Emotion, Siemens, Switzerland), respectively. Imaging revealed a large, multilobular, mineralized osteodestructive mass measuring 5 cm × 6 cm × 8 cm, arising from the occipital and parietal bones of the skull. The mass exhibited a coarse, granular appearance and caused significant mass effect, displacing and effacing most of the cerebellum and occipital lobes. Transtentorial herniation and brain edema were also evident. Additionally, MRI findings suggestive of secondary spinal cord edema were observed in the cranial cervical spine. Based on the comprehensive imaging features, multilobular osteochondrosarcoma was considered the most likely diagnosis.

To assess for metastasis, CT scans of the thorax and abdomen were performed, revealing no evidence of distant spread. Notably, careful evaluation of post contrast imaging focused on the skull presence/absence of contrast enhancement within large venous sinuses and their patency. These images demonstrated compression of the caudal portion of the dorsal sagittal sinus by the mass.

### Preoperative virtual surgical planning

The patient’s CT scans were exported as Digital Imaging and Communications in Medicine (DICOM) format and imported into a medical imaging software (Materialize Mimics Innovation Suite, version 24.0, Materialize NV, Leuven, Belgium), where the skull bones and the tumor extension were demarcated. After the segmentation process, the bone and tumor surface geometry was saved as a Standard Tessellation Language (STL) file. Subsequently, the patient-specific cutting guides and the cranial implants were designed using computer-aided design modeling software (Geomagic Freeform, version 2021, 3D Systems, Rock Hill, South Carolina, United States).

**Figure 1:**
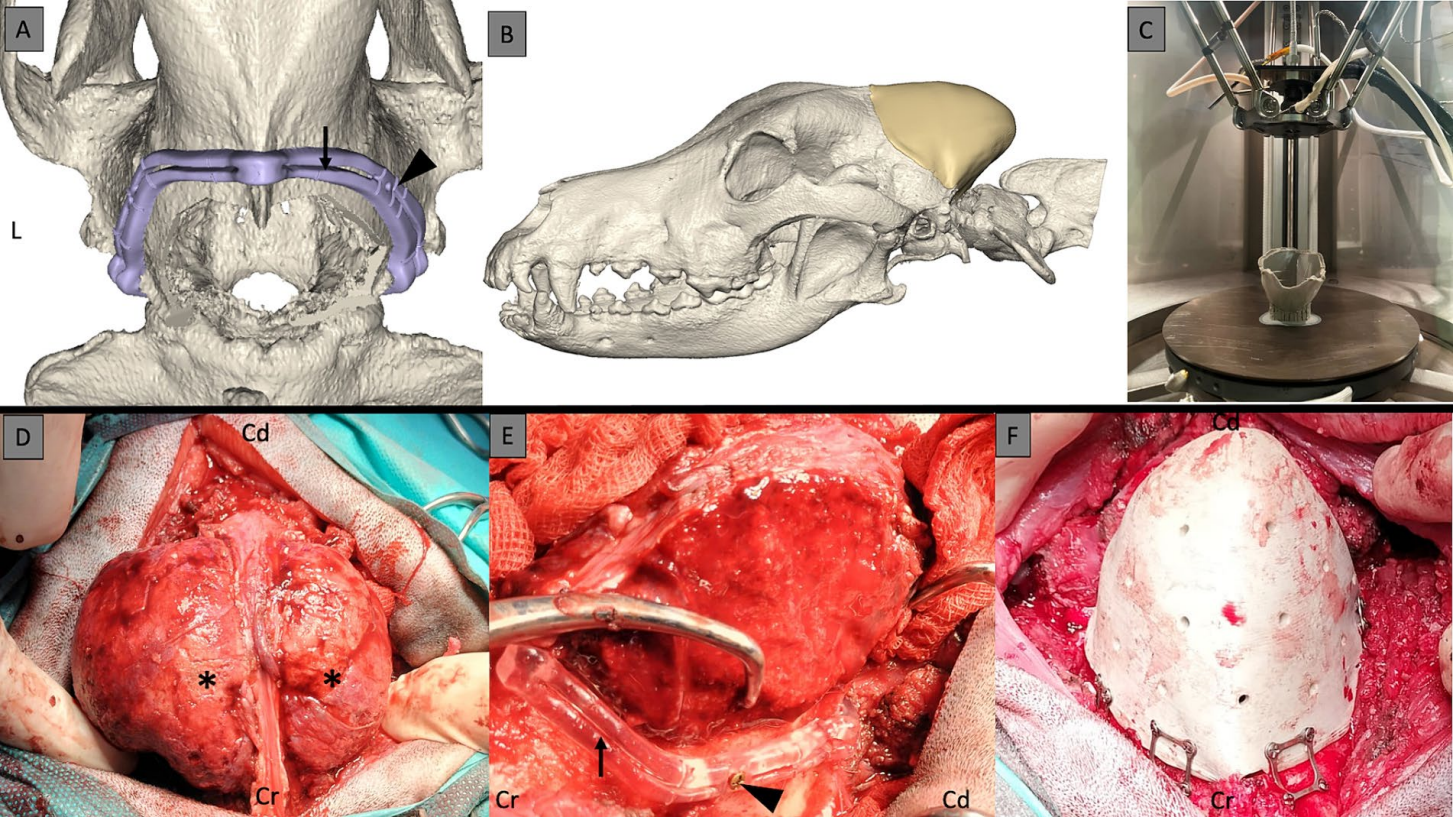
3D surgical planning of the cutting guides with the groove (black arrow) for the blade of the bone scalpel and the holes for the fixation screws (black arrowhead) **(A)**. 3D surgical planning with the skull prosthesis in place to fit exactly the craniotomy line **(B)**. 3D printing of the PEEK implant **(C)**. Multilobular osteochondrosarcoma (MLO) intraoperatively with a bilobed shape (black asterisks) **(D)**. MLO stripped of its masses to allow for placement of the cutting guides with craniotomy grove (black arrow) and the fixation screws (black arrowhead) **(E)**. 3D-printed PEEK skull implant after craniectomy and attached to the skull with screws and plates **(F)**. Cr, cranial; Cd, caudal; L, left.

Bilateral cutting guides, tailored for both the right and left sides of the cranial tumor extent, were meticulously designed. These guides were planned with specific features to accommodate the resection procedure effectively, integrating craniotomy grove to fit the Misonix BoneScalpel^®^ (MBS) blade and fixation screw placements. The left and right cutting guides possessed a V-shaped interlocking feature to facilitate easier assembly, ensuring accurate and effective guidance during the surgical procedure. For the skull-specific craniotomy guide, the craniotomy line was first drawn 5 mm away from the skull tumor margins on the CAD software in order to balance the likelihood of clear margins and preservation of important anatomical landmarks (such as the occipital condyles). On each side of that line, two guiding walls were then designed. This led to a guide composed of two walls and a craniotomy groove in which the blade of the bone scalpel would slide in. In order to secure the guide to the skull, holes for fixation screws were added in the design (Figures 1A,E). These cutting guides were subsequently fabricated through Stereolithography (SLA) 3D printing technology utilizing biocompatible materials (BioMed Clear, Formlabs, Ohio, United States). In addition, a 3D model of the dog’s occipital skull template was fabricated using a material-extrusion 3D printer [MakerBot PLA Filament (true white), MakerBot Replicator+, MakerBot Industries, Brooklyn, New York, United States]. This step evaluated the fit of the surgical cutting guide and the cranial implant. By utilizing this template model, the surgical team could assess the precise alignment and positioning of the guide and implant in relation to the patient’s skull anatomy, ensuring optimal surgical outcomes.

To produce the 3D-printed PEEK patient-specific cranial implant, a material extrusion-based 3D printer (Kumovis R1.2, Kumovis GmbH, Munich, Germany) was employed. The STL file of the cranial implant design was imported into the 3D printer’s slicing software (Simplify3D 4.1.1, Cincinnati, United States), where specific printing parameters for PEEK were chosen. Utilizing a 1.75 mm PEEK filament (Evonik Vestakeep^®^i4 3DF, Evonik Industries AG, Essen, Germany), the printer fabricated the cranial implant, with the sliced G-code file subsequently transferred to the printer (Figure 1C). After printing the cranial implant, the raft and support structures were manually removed using rotary tools, with no additional post-processing steps. Following fabrication, the PEEK cranial implant underwent autoclave sterilization, while the guide and skull template were sterilized with H_2_O_2_ according to standard protocols.

### Surgical management

For the pre-operative management, a two-week course of corticosteroids (prednisolone, 1 mg/kg/day, PO) was administered pre-operatively to reduce brain edema. Figure 1 shows the pre-and post-surgery planning with the cutting guides and prosthesis.

The surgery was performed under general anesthesia with the patient in sternal recumbency. Following aseptic preparation, a midline skin incision was made between the orbits to the caudal portion of C2. The temporalis muscles were reflected bilaterally to expose the skull. In the occipital region, the superficial and deep cervical musculature were dissected to elevate the musculature from the occipital bone, C1 dorsal arch, and C2 spinous process. Wet gauze covered and moistened the exposed musculature throughout the surgery. The entire skull was exposed from one zygomatic arch to the other and caudally to the C1 transverse processes. The 3D-printed skull template and cutting guides guided the extent of exposure.

A Misonix BoneScalpel^®^ (MBS) with 3D-printed cutting guides facilitated the craniotomy. After clearing residual soft tissue from the contact points, the guides were secured with 3 mm self-cutting skull screws. The bilobed MLO necessitated initial excision of its outer portion before guide placement. The MBS with a 20 mm Blade Blunt (MXB-20) facilitated near-bloodless tumor resection. Following this, a lateral craniotomy was performed using the cutting guides. Full-thickness osteotomy was achieved, except near the dorsal sagittal and transverse sinuses, where a Ø4.4 Diamond Shaver (MXB-S3) was utilized to prevent their transection. The lateral craniotomy was extended dorsally, caudally, and ventrally to achieve a lateral osteotomy while preserving the midline and caudal vascular regions. Bone flaps were carefully elevated from the dura mater bilaterally using a Freer periosteal elevator. Bipolar cautery on low settings controlled bleeding (10 Watt maximum, ICC 350, ERBE, Tubingen Germany) Tactile feedback aided in identifying the inner cortical layer during bone removal with the MBS and blade blunt. The diamond shaver connected the two lateral craniectomies at their cranial and caudal contact points over the dorsal sagittal and transverse sinuses, respectively. With complete craniotomy achieved, the intracranial MLO was gradually elevated dorsally and caudally using hand traction and a Freer periosteal elevator to detach the dura mater safely from the abnormal bone. Moderate hemorrhage from the left transverse sinus was controlled with obliteration with bone wax.

The 3D-printed patient-specific PEEK cranial implant was positioned over the craniectomy defect and secured with 3.0 mm self-drilling screws and skull plates (Matrix Neuro Plates, 0.4 mm thickness, Synthes, Zuchwil, Switzerland). While the fit was deemed satisfactory, a slight imperfection was noted in the most caudal portion of the occiput due to friable bone.

**Figure 2:**
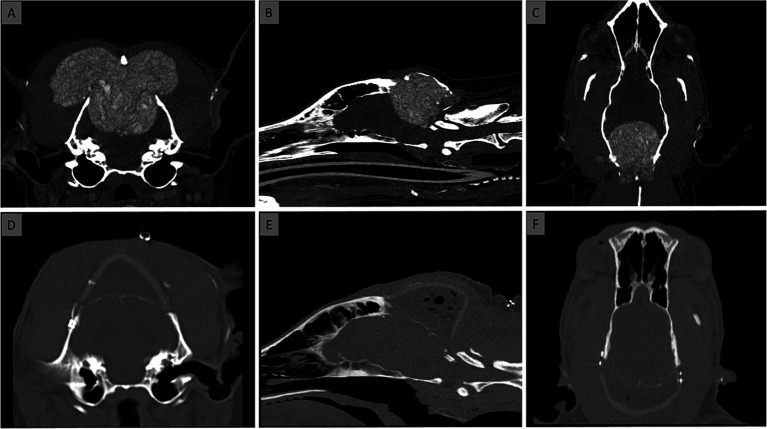
Computed Tomography [CT; transverse **(A,D)**, sagittal **(B,E)**, dorsal **(C,F)** views] at the time of diagnosis **(A–C)** and after placement of the prosthesis **(D–F)**. No dorsal sagittal sinus was visible at the level of the MLO on post contrast CT and MRI.

Polypropylene mesh (Prolene, Ethicon, Somerville, NJ) was used for neck muscle reconstruction. The mesh was left intact in the cervical portion but divided medially to allow expansion over each skull side. The mesh was cut to the deeper cervical musculature dorsally and covered with the superficial muscle layer. The mesh was then attached to the bilateral masseter muscle fascia using polydioxanone sutures (PDS II, Ethicon, Somerville, NJ). The mesh was not attached to the skull implant to prevent traction. Dead space between muscle layers was closed with interrupted sutures. Subcutaneous tissue and skin were closed with absorbable sutures and skin staples, respectively. Figure 2 shows pre- and post-operative CT.

Intraoperatively, the patient received cefazolin (22 mg/kg, IV), methylprednisolone succinate (10 mg/kg, IV), and mannitol (1 g/kg IV over 20 min). The surgical procedure lasted 4 h. Postoperative pain management included buprenorphine for 3 days, followed by a combination of prednisolone (1 mg/kg/day, PO), cefalexin (20 mg/kg, twice daily, PO), and gabapentin (10 mg/kg three times per day, PO) for 3 weeks. The dog was discharged on postoperative day 3 with neurological signs unchanged from pre-surgery. Histopathological examination of the resected bone confirmed a Grade I MLO according to the Dernell classification (2). Although the margins were free of tumor, potential recurrence was acknowledged due to the tumor’s proximity to the resection margins.

### Follow-Up

Regular telephone consultations were conducted to monitor the dog’s clinical and neurological status. The eight-week follow-up revealed that the dog had a residual right pelvic limb proprioceptive deficit, while other neurological examination results had returned to normal. The owner observed marked improvements in the dog’s gait, vision, behavior, and mentation within the first 3 weeks after the surgery. A computed tomography (CT) scan carried out 8 weeks post-surgery revealed a periosteal reaction at the cranial interface between the skull and the prosthesis and on the ventral aspect of the occipital craniectomy. There were no signs of implant rejection. The dog commenced chemotherapy 10 weeks following the surgery, with a regimen including Cyclophosphamide at 12.5 mg/day PO, Furosemide at 20 mg/day PO, Thalidomide at 50 mg/day PO, and Piroxicam at 15 mg/day PO as needed.

At 53 weeks post-surgery, the dog exhibited symptoms of apathy and hematochezia, but an abdominal ultrasound did not reveal any abnormalities. The dog received symptomatic inpatient treatment for 3 days, and chemotherapy was halted for 4 weeks due to suspected gastrointestinal adverse effects from the medication.

Seventy-nine weeks after the surgery, the dog developed coughing and vomiting, leading to a diagnosis of aspiration pneumonia and megaesophagus. Tests for Acetylcholine receptor antibodies, TSH, T4, and cortisol levels were all within normal limits. The dog was treated symptomatically with antibiotics for 3 days as an inpatient and was then discharged with a suspicion diagnosis of acquired idiopathic megaesophagus. Regrettably, the dog died at home. Ultimately, the dog’s post-surgical survival time was 18 months.

## Discussion

This case report demonstrates the successful application of a patient-specific 3D-printed polyetheretherketone (PEEK) cranial implant for cranioplasty following extensive tumor resection in a dog.

The selection of PEEK offered many reported advantages, including its robustness, lightweight nature and comfort for the animal. Moreover, PEEK implants are known for their biocompatibility and compatibility with imaging studies, thus ensuring diagnostic accuracy. Notably, they can withstand sterilization procedures while maintaining their structural integrity (17). Furthermore, PEEK cranial implants demonstrate sufficient dimensional accuracy and possess mechanical properties similar to cortical bone, essential for ensuring effective reconstruction and safeguarding anatomical cranial structures (18, 19). Nevertheless, it is essential to acknowledge the limitations associated with PEEK cranial implants. While 3D-printed PEEK cranial implants offer numerous benefits, these tend to be more costly compared to standard stock implants. Additionally, PEEK lacks osteointegration capabilities, hindering its ability to seamlessly integrate with surrounding bone tissue (20).

Conversely, other biomaterials commonly used in cranioplasty present their own set of advantages and drawbacks. For instance, polymethyl methacrylate (PMMA), although cost-effective and readily available, poses challenges in molding and adapting to complex cranial defects, potentially prolonging surgery time and increasing infection risks (21).Titanium mesh, known for its excellent biocompatibility and favorable mechanical strength, is prone to infection rates and deformation under trauma and can cause significant imaging artifacts (22). Recent studies also suggest a higher risk of implant failure with other biomaterials compared to PEEK cranial implants (23). Hydroxyapatite, while promoting bone growth, is brittle and may lead to fractures before complete integration (24), particularly in adult animals.

This case report highlights the potential advantages of using 3D-printed technology for cranioplasty: (1) improved accuracy and efficiency: Patient-specific implants ensure a precise fit, minimizing the risk of margin errors and facilitating reconstruction. (2) Enhanced visualization and planning: 3D-printed models of the skull and tumor can aid preoperative planning and improve intraoperative decision-making. (3) Reduced surgery time and potential complications: Precise cutting guides can streamline the craniotomy process, potentially reducing surgical time and associated risks.

Limitations and future directions: while this report demonstrates the successful application of this technology in a single case, further research is needed to evaluate its broader efficacy and long-term outcomes in a larger population. Additionally, considerations regarding cost-effectiveness and accessibility of 3D printing technology need to be addressed for wider adoption in veterinary medicine.

Overall, this case report contributes to the growing body of evidence supporting the potential benefits of 3D-printed patient-specific implants and cutting guides for cranioplasty in veterinary patients.

## Data availability statement

The original contributions presented in the study are included in the article/supplementary material, further inquiries can be directed to the corresponding author.

## Ethics statement

Ethical approval was not required for the studies involving animals in accordance with the local legislation and institutional requirements because this is a retrospective case report based on clinical and imaging findings. Owner did give verbal consent for use of CT and surgery data for publication. Written informed consent was not obtained from the owners for the participation of their animals in this study because this is a retrospective case report based on clinical and imaging findings.

## Author contributions

MH: Writing – original draft, Writing – review & editing, Conceptualization. NS: Writing – original draft, Writing – review & editing, Conceptualization, Methodology, Software, Supervision, Validation, Visualization. CB: Writing – original draft, Writing – review & editing, Investigation, Validation. SH: Writing – original draft, Writing – review & editing, Investigation, Supervision, Validation. AO: Writing – original draft, Writing – review & editing, Formal analysis, Supervision, Validation. MM: Writing – original draft, Writing – review & editing, Investigation, Methodology, Software. MB: Writing – original draft, Writing – review & editing, Investigation, Methodology, Software. FT: Writing – original draft, Writing – review & editing, Supervision, Validation. JG: Writing – original draft, Writing – review & editing, Conceptualization, Formal analysis, Investigation, Methodology, Project administration, Resources, Supervision, Validation, Visualization.
